# A landscape assessment of the use of patient reported outcome measures in research, quality improvement and clinical care across a healthcare organisation

**DOI:** 10.1186/s12913-023-09050-1

**Published:** 2023-01-27

**Authors:** David A. Snowdon, Velandai Srikanth, Richard Beare, Lucy Marsh, Emily Parker, Kim Naude, Nadine E. Andrew

**Affiliations:** 1 National Centre for Healthy Ageing, Melbourne, VIC Australia; 2 Peninsula Clinical School, Central Clinical School, Monash University, Melbourne, VIC Australia; 3 Academic Unit, Peninsula Health, Frankston, VIC Australia

**Keywords:** Patient reported outcome measures, Implementation science, Value-based healthcare

## Abstract

**Background:**

Patient reported outcome measures (PROMs) can be used by healthcare organisations to inform improvements in service delivery. However, routine collection of PROMs is difficult to achieve across an entire healthcare organisation. An understanding of the use of PROMs within an organisation can provide valuable insights on the purpose, scope and practical considerations of PROMs collection, which can inform implementation of PROMs.

**Methods:**

We used multiple research methods to assess the use of PROMs in research projects, data registries and clinical care across a healthcare organisation from January 2014 to April 2021. The methods included an audit of ethics applications approved by the organisation’s human research ethics committee and registries which the health organisation had contributed data to; a literature review of peer-reviewed journal articles reporting on research projects conducted at the organisation; and a survey of health professionals use of PROMs in research projects, data registries and clinical care. The scope of PROMs was determined by classifying PROMs as either ‘specific’ to a particular disease and/or condition, or as a ‘generic’ measure with further classification based on the health domains they measured, using the World Health Organization International Classification Framework. Practical considerations included mode and timing of PROMs administration. Data were described using frequency and proportion.

**Results:**

PROMs were used by 22% of research projects (*n* = 144/666), 68% of data registries (*n* = 13/19), and 76% of clinical specialties in their clinical care (*n* = 16/21). Disease specific PROMs were most commonly used: 83% of research projects (*n* = 130/144), 69% of clinical registries (*n* = 9/13), and 75% of clinical specialties (*n* = 12/16). Greater than 80% of research projects, clinical registries and clinical specialties measured health domains relating to both body impairments and participation in daily life activities. The most commonly used generic PROM was the EQ-5D (research projects *n* = 56/144, 39%; data registries *n* = 5/13, 38%; clinical specialties *n* = 4/16, 25%). PROMs used in clinical care were mostly paper-based (*n* = 47/55, 85%).

**Conclusions:**

We have elicited information on the use of PROMs to inform a health organisation wide implementation strategy. Future work will determine clinician and patient acceptability of the EQ-5D, and co-design a system for the collection of PROMs.

**Supplementary Information:**

The online version contains supplementary material available at 10.1186/s12913-023-09050-1.

## Background

With rising healthcare costs, there is mounting pressure on healthcare organisations to provide services that produce outcomes that are meaningful to patients at the lowest possible cost [1]. Value-based healthcare is a healthcare delivery model in which providers are incentivised to maximise patient relevant outcomes relative to the amount or type of care provided [1, 2], with the aims of improving population health, enhancing patient satisfaction, and reducing costs [2, 3]. At the core of value-based healthcare is the dependence on reliably measuring outcomes that capture what matters to patients [1].

Patient reported outcome measures (PROMs) are self-reported measures of a person’s health status or health related quality of life [4]. PROMs can be classified as generic or specific to a disease or health condition [5–7]. They also measure different aspects of health, including functional impairments, symptoms and limitations in execution of activities and/or participating in life situations [8]. PROMs are used in: clinical care to support provider-patient decisions; quality improvement to evaluate healthcare provider performance and guide initiatives to improve healthcare; and research to measure population health and evaluate the effectiveness and cost utility of healthcare innovations/interventions [3, 4, 9, 10].

For PROMs to be effectively used in value-based healthcare, they need to be routinely collected; ensuring that adequate data is captured (i.e. high PROM completion rates) to provide an accurate and unbiased indication of healthcare performance [4, 11]. However, large scale routine collection of PROMs across an entire health organisation or population is difficult [11, 12]. As a consequence, routine collection of PROMs to date has been limited to individual clinics [13], specific clinical specialties or discreet conditions [14–16]. Barriers to routine collection include responder burden for patients, health professionals’ perception that PROMs lack clinical utility, the time and resources required to collect PROMs from an organisational perspective, and limited ability to share PROM data between services and clinical specialties [11, 17]. Also, a challenge to the implementation of routine collection of PROMs across a health organisation is the variety of services and clinical specialties within the organisation, which all have different needs and preferences. To overcome these barriers it is recommended that an evidence-based systematic approach, taking into account the needs of multiple stakeholders, is used when implementing routine collection of PROMs [13].

To date, there has been significant variability in implementation approaches in the routine collection of PROMs across health services (i.e. implementation at multiple hospitals or community centres). Most approaches have involved engagement of exclusively clinicians [18, 19], exclusively patients [20], or both clinicians and patients during a trial period of PROMs collection (i.e. feasibility trial) [17, 21, 22]. Methods of engagement have included training [18], surveys [22], qualitative focus groups/workshops [20, 21], and quality improvement processes [19]. However, there are few examples of engagement of stakeholders during the pre-implementation phase (i.e. planning) when deciding which PROM(s) to collect and how to collect them. Where stakeholders have been engaged in the pre-implementation phase, engagement has predominantly been with clinicians and has lacked the detail in reporting that is required for replication [19, 20, 22]. Engagement during pre-implementation has typically aimed to establish what PROMs have historically been used by clinicians via survey rather than objective measures of use [19, 22]. Despite variability in approach to implementation, outcomes have consistently shown poor PROM completion rates at follow up [17, 20, 23–25], and variability between health services [17, 19, 20, 23, 24]. Hence, there is a need for further research to establish how best to plan the implementation of routine collection of PROMs across health services to maximise uptake.

Across a healthcare organisation it is likely that the health services and clinicians working within these services would have had experience using PROMs. Therefore, an essential precursor to large scale implementation of PROMs collection is to understand the existing context from a range of perspectives, considering the different reasons for collection (e.g. clinical care, quality improvement or research). [11, 26, 27] Knowledge of the historical and current use of PROMs within a healthcare organisation can provide valuable insights on the purpose, scope and practical considerations of PROM use. Understanding the diversity in PROM use across an entire healthcare organisation, spanning multiple clinical services and specialties, has the potential to provide critical information to guide selection and implementation of PROM(s), whilst maximising value from the perspective of multiple stakeholders.

The aim of this study was to develop and apply a landscape assessment to better understand the current use of PROMs across an entire healthcare organisation. In doing so we aimed to describe: (1) the scope of PROMs used across the organisation; (2) the purpose for which the PROMs were being collected; (3) how the scope of PROMs use differed based on purpose and clinical specialties; and (4) the method of collection, including mode, timing and time taken.

## Methods

### Study design

**Fig. 1:**
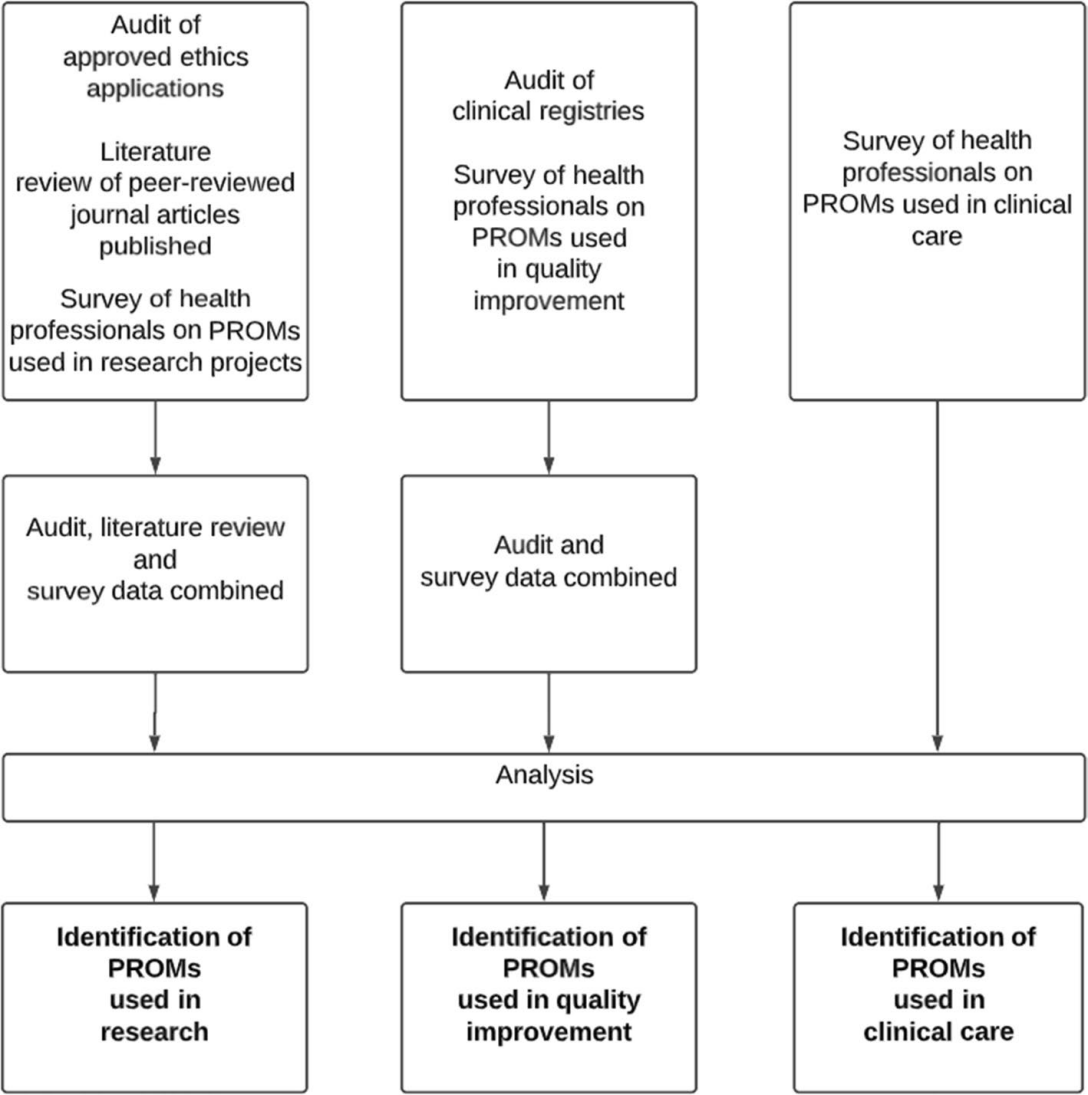
Study design: Landscape assessment of the use of Patient Reported Outcome Measures from January 2014 to April 2021

PROMs are predominantly used in three main contexts: outcomes-based research, quality improvement and clinical practice. [4] Therefore, to assess the use of PROMs across the health organisation we used several study designs to address each of these purposes (Fig. 1). First, to assess the use of PROMs for research we conducted: (i) an audit of ethics applications approved by the organisation’s human research ethics committee, (ii) a literature review of peer-reviewed journal articles reporting on projects conducted at the organisation, and (iii) a survey of health professionals use of PROMs in research projects. Second, to assess the use of PROMs for quality improvement, we conducted: (i) an audit of registries which the health organisation had contributed data to, and (ii) a survey of health professionals use of PROMs in clinical registries. Third, to assess the use of PROMs for clinical care, we conducted a survey of health professionals use of PROMs in clinical care. We assessed the use of PROMs in research, quality improvement and clinical care over a seven-year period from January 2014 to April 2021. Approval for this study was obtained from the Peninsula Health Human Research Ethics Committee (LNR/73731/PH-2021).

### Setting

This study is part of a broader body of work undertaken in the establishment of the National Centre for Healthy Ageing Health Research Data Platform, a collaboration between Monash University and Peninsula Health in Victoria, Australia. The Platform curates high quality data from hospital, government and community-based healthcare systems across a geographic region to support research, clinical care, evidence-based healthcare improvement and research for high priority areas of health, related to ageing. Central to the Data Platform is the implementation and integration of a system for routine collection of PROMs across an entire healthcare organisation with a view to expanding collection to the entire region.

Peninsula Health is a publicly funded healthcare organisation that services over 300,000 people living in the Shire of Mornington Peninsula. [28] It provides hospital and community-based healthcare services across a range of different specialties, including emergency care, intensive care, aged care, obstetrics and gynecology, oncology, and mental health. The health organisation consists of four hospitals, including one tertiary hospital, and more than 10 community healthcare sites.

### Overarching methodological framework

**Fig. 2:**
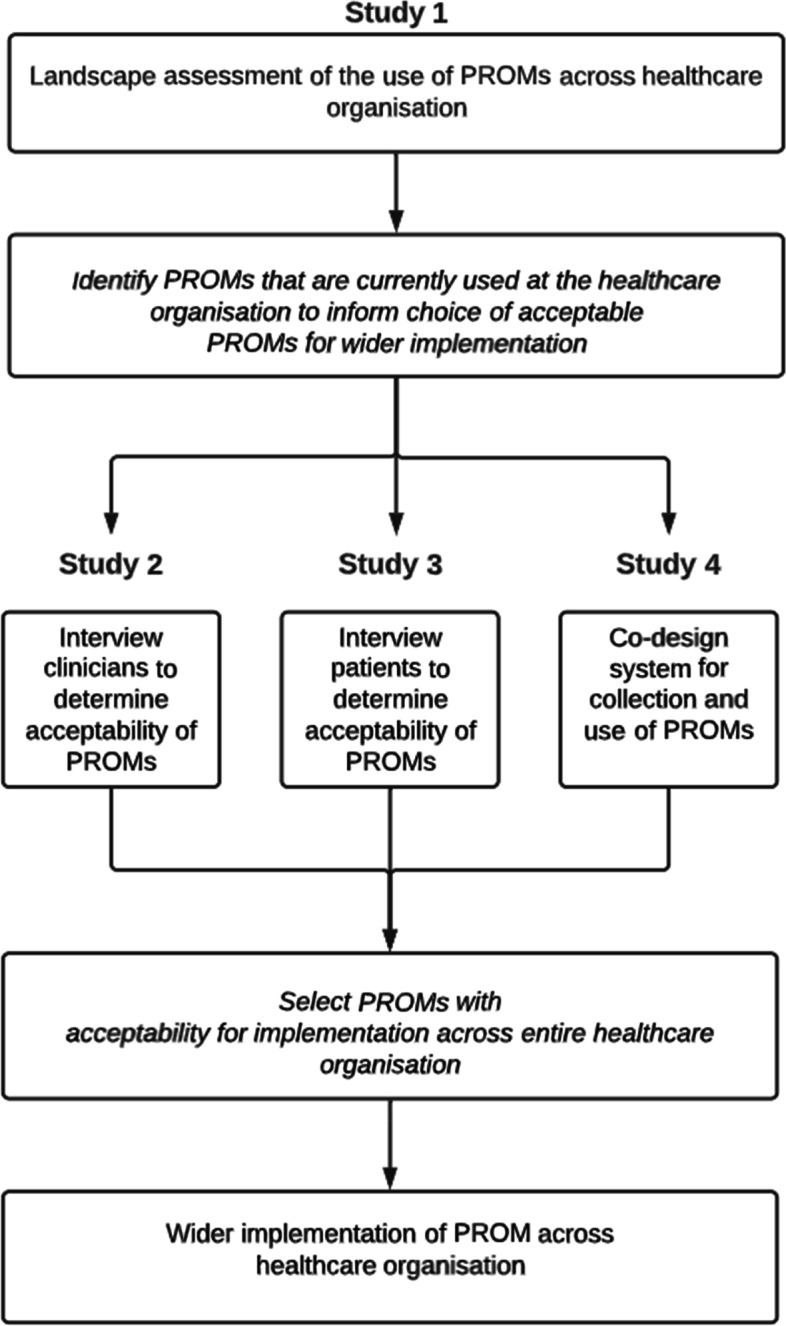
Planning implementation of routine collection of Patient Reported Outcome Measures across Peninsula Health

Recommended steps for planning implementation of routine collection of PROMs include determining: (1) the purpose of collecting the PROM; (2) the scope (e.g. generic vs. specific, what aspects of health it measures) of the PROM; (3) the practical considerations (e.g. mode of administration, timing of administration); (4) the patient and clinician acceptability of the PROM; and (5) the measurement properties of the PROM [12, 26, 27]. To address these steps we propose a program of work consisting of four studies (Fig. 2), of which this is the first. In this first study we have sought to gain a contextual understanding of the use of PROMs across the healthcare organisation to establish the purpose, scope and practical considerations of routine PROMs collection. The second and third studies will involve gaining the perspectives of patients and clinicians of the acceptability of PROMs identified during the landscape assessment, and feasibility of routine collection of these items. The PROMs selected for implementation across the organisation will need to demonstrate clinician and patient acceptability, feasibility of routine collection, and validity and reliability in measuring health status in a broad range of populations and settings. The fourth study will engage with patients to co-design a system for collection and use of PROMs across the healthcare organisation.

### Data collection

#### Part 1 – Research

- (i) Audits
  An audit of ethics applications, approved by the organisation’s human research ethics committee between January 2014 and April 2021, was conducted to identify research projects using established PROMs and the PROMs used in each project. A report was generated by the office for research containing all eligible project applications and related documents (i.e. all project applications that were approved by the committee during the designated time period). Projects with PROM documents attached to their application as outcome measures were considered to have included PROMs. Where a project did not have a PROM document attached to their application, a de-identified version of the ethics application was reviewed by the research team to determine whether the project used a PROM as an outcome measure.
- (ii) Literature review
  A literature review of peer-reviewed journal articles reporting on projects conducted at the organisation was conducted to complement the audit of approved projects. Pubmed, Embase (Ovid), Cumulative Index to Nursing and Allied Health Literature [CINAHL] (EbscoHost) and PsychINFO (Ovid) databases were searched from January 2015 to April 2021. The search was performed from 2015 as it was not expected a project that commenced in 2014 would publish in the same year. Search terms used included the names of Peninsula Health hospitals and community health centres (Additional file 1). Articles were screened to determine if the projects they report on 1) were conducted at Peninsula Health and 2) administered a PROM (i.e. inclusion criteria). Two researchers independently screened articles by title and abstract. Articles that were not definitely excluded on title and abstract were retrieved for detailed examination. Two researchers independently screened full text articles with discussion ensuing to reach a consensus on whether the project met inclusion criteria.
- (iii) Survey
  Responses from the clinician survey in Part 3 were used to validate the results obtained from the ethics audit and literature review.

#### Part 2 – Quality improvement

- (i) Audit
  An audit of registries for which the health organisation contributed data was conducted to identify registries that collected PROMs and the individual PROMs collected in each registry. A report of all registries with ethical approval for routine data collection was obtained from the health organisation’s office for research. Data items collected for each registry were obtained by the research team from registry websites and audited for PROMs.
- (ii) Survey
  Responses from the clinician survey in Part 3 were used to validate the registry audit results.

#### Part 3 – Clinical care

- (i) Survey
  A survey of health professionals use of PROMs in their clinical care was conducted to identify the clinical specialties that use PROMs, and the actual PROMs used. They were also asked to provide information on mode (e.g. web-based, paper-based, telephone) and timing of PROMs collection. An invitation to participate in the study, including a link to the electronic survey, was emailed to managers/program directors of all clinical specialty areas within the health organisation, who then forwarded the email to the senior health professional(s) working under their remit. Senior health professionals included medical, nursing and allied health professionals. A reminder email was sent two weeks following the initial invitation. All participants provided informed consent prior to completing the survey. Please refer to Additional file 2 for survey questions.

### Categorising PROMs

#### Purpose of collecting PROMs

The reason for collecting each PROM was categorised into the three common purposes for use in healthcare: research; quality improvement; and clinical care [3, 4, 9, 10]. We further categorised PROMs used for research based on whether they were used as an endpoint in the study or as an exploratory outcome; and PROMs use for clinical care based on whether they were used to inform patient-provider decisions at the individual patient-level or to inform care at the service-level.

#### Scope of PROMs

To define the scope of PROMs in this study, we categorised PROMs into generic and specific groupings [26]. Generic PROMs included those that measured aspects of health status which are common to most patients, allowing comparison between patients with a wide variety of conditions. [5–7] Specific PROMs included both disease and condition specific PROMs, which measured aspects of health status of a particular disease or patient group/condition [6, 7].

PROMs were also grouped by the aspect of health they measured, as defined by The International Classification of Functioning, Disability and Health (ICF) [8]. PROMs were categorised as measuring only ‘body structure and function’, only ‘activities and participation’, or both ‘body structure and function’ and ‘activities and participation’ [29]. ‘Body function and structure’ refers to the physiological functions of body systems and anatomical parts of the body (e.g. organs, limbs); ‘activities’ refers to the execution of tasks or actions (e.g. walking); and ‘participation’ refers to involvement in a life situation (e.g. paid or voluntary work) [8]. Our approach to combining the ‘activities’ and ‘participation’ concepts is consistent with ICF user guidelines [29].

The scope of each PROM was independently determined by two researchers. Disagreement between researchers was resolved by discussion until consensus was reached, or by consulting a third project team member.

#### Practical considerations of PROMs collection

The practical considerations that were included in our landscape assessment were the mode of administration, timing of administration and the time taken to administer the PROM [16, 30–32]. Mode was categorised as either paper-based or electronic, and face-to-face, mail, or email/link. Timing was categorised as prior to admission, on admission, daily during admission, on discharge, 1 to 6 months follow-up, > 6 months follow up, or as required. Time taken to administer PROMs was categorised as ≤ 5 min, 6–10 min, or ≥ 10 min.

### Data analysis

All data were analysed descriptively. Data from multiple sources were combined to produce a single result for research (i.e. audit, literature review and survey), and quality improvement (i.e. audit and survey).

To assess the purpose of PROMs collection, the frequency and proportion of research projects, registries and clinical specialties using PROMs was calculated. We also calculated the frequency and proportion of research projects that used PROMs as an endpoint in the study or as an exploratory outcome; and the frequency and proportion of clinical specialties that used PROMs to inform patient-provider decisions at the individual patient-level or to inform care at the service-level.

To assess the scope of PROMs the frequency and proportion of research projects, registries and clinical specialties using each type of PROM (i.e. ‘generic’ vs. ‘specific’) and measuring each aspect of health (i.e. ‘body structure and function’ vs. ‘activities and participation’ vs. ‘all aspects’) were calculated. Sub-group analyses were conducted to determine the frequency of each generic and specific PROM used for research, registries and clinical care. Specific PROMs were stratified by clinical specialty.

To assess the practical considerations of PROMs collection the method of collection, including mode, timing and time taken to administer PROM in clinical care were described using frequency and proportion of PROMs used. Timing of PROMs collection was stratified by hospital and community-based services.

## Results

**Fig. 3:**
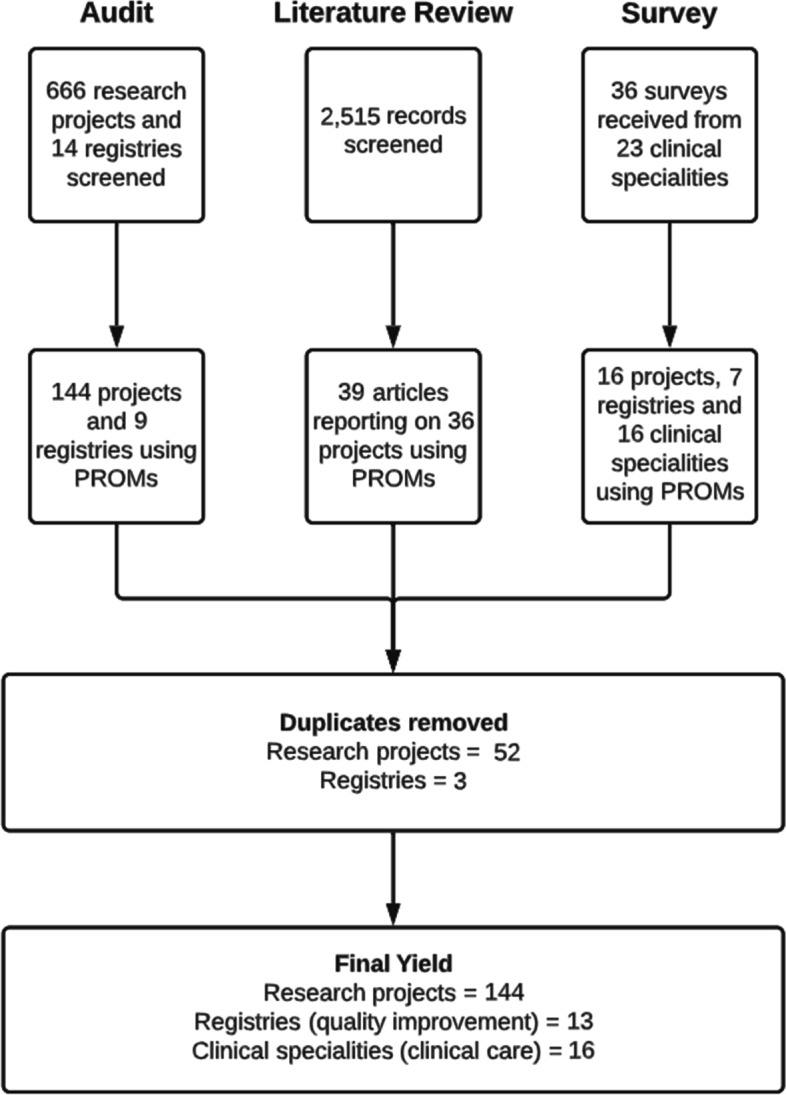
Flow chart for identification of Patient Reported Outcome Measures used in research, quality improvement and clinical care

Please refer to Fig. 3 for a flow chart outlining the identification of projects, registries and clinical specialties using PROMs for research, quality improvement and clinical care, respectively. Results from the literature search can be found in Additional file 3.

### Purpose and scope of PROMs

#### Part 1—Research

**Fig. 4:**
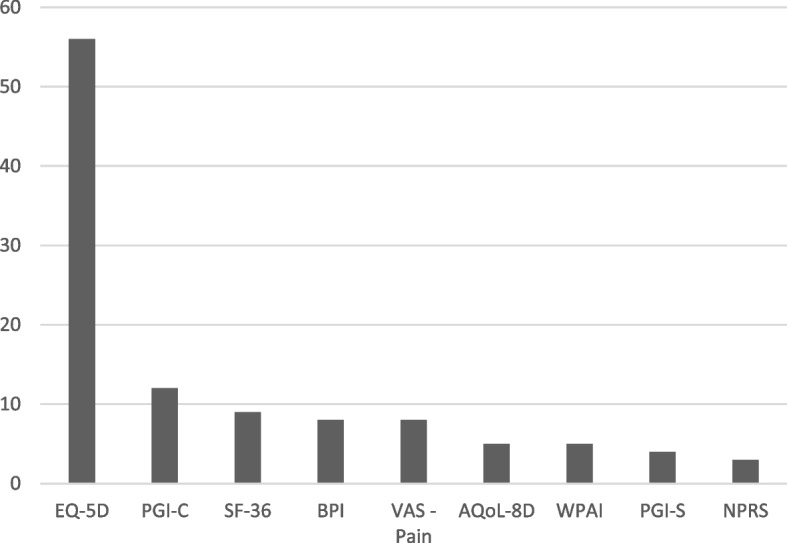
Most frequently used generic Patient Reported Outcome Measures in research projects. Legend: PGI-C: Patient Global Impression of Change Scale; SF-36: 36-Item Short Form Survey; BPI: Brief Pain Inventory; VAS: Visual Analogue Scale; AQoL-8D: Assessment of Quality of Life-8 Dimensions; WPAI: Work Productivity and Activity Impairment Questionnaire; PGI-S: Patient Global Impression of Severity Scale; NPRS: Numerical Pain Rating Scale

Of the 666 research projects identified 144 (22%) used PROMs. Of these, 115 (80%) used a PROM as an endpoint in a study and 29 (20%) as an exploratory outcome. In total, there were 171 PROMs used, 130 (76%) specific and 41 (24%) generic. Most projects measured both body function and activity/participation (93%) and used a specific PROM (83%), with 48% using both a generic and specific PROM (Table 1). The most frequently used generic PROM was the EQ-5D, which was used in 56 projects (60% of projects using a generic PROM). Of these, 31 projects used the five-level version (EQ-5D-5L) and 25 projects used the three-level version (EQ-5D-3L). The Patient Global Impression of Change Scale was the next most commonly used generic PROM (*n* = 12, 13% of projects using a generic PROM) (Fig. 4). Please refer to Additional files 4 and 5 for a summary of the generic and specific PROMs used for research.

**Table 1:** Scope of patient reported outcome measures used for research, quality improvement and clinical care

Scope	Research	Quality improvement	Clinical care
	*n of projects (%)*	*n of registries (%)*	*n clinical specialties (%)*
**Total**	144	13	16
**Type**			
Only generic	24 (17)	4 (31)	4 (25)
Only specific	51 (35)	6 (46)	8 (50)
Both	69 (48)	3 (23)	4 (25)
**WHO ICF Domain**			
Only body function	9 (6)	0 (0)	3 (19)
Only activity/participation	1(1)	0 (0)	0 (0)
Both	134 (93)	13 (100)	13 (81)

*WHO ICF* World Health Organization International Classification Framework

#### Part 2—Quality improvement

**Fig. 5:**
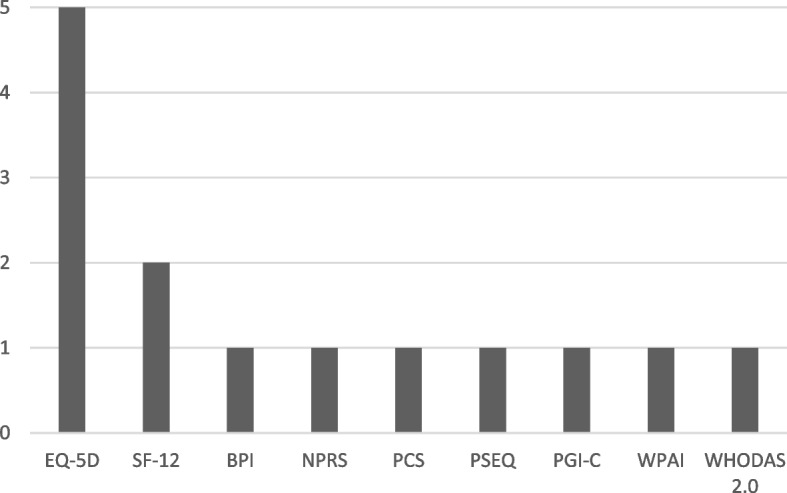
Most frequently used generic Patient Reported Outcome Measures in quality improvement registries. Legend: BPI: Brief Pain Inventory; PCS: Pain Catastrophising Scale; PGI-C: Patient Global Impression of Change Scale; PSEQ: Pain Self Efficacy Questionnaire; NPRS: Numerical Pain Rating Scale; SF-12: 12-Item Short Form Survey; WPAI: Work Productivity and Activity Impairment Questionnaire; WHODAS 2.0: World Health Organization Disability Assessment Schedule 2.0

Of the 19 registries identified 13 (68%) used PROMs for quality improvement. In total, there were 21 PROMs used, 11 (52%) specific and 10 (48%) generic. All registries measured both body function and activity/participation and most used a specific PROM (69%), with 23% using both generic and specific PROMs (Table 1). The most frequently used generic PROM was the EQ-5D (*n* = 5 registries, 71% of registries using generic PROMs) with 3 using the EQ-5D-3L and 2 using the EQ-5D-5L. The 12-Item Short Form Survey was the next most commonly used generic PROM (*n* = 2, 29% of registries using a generic PROM) (Fig. 5). Please refer to Additional files 4 and 5 for a summary of the generic and specific PROMs used for quality improvement.

#### Part 3—Clinical care

**Fig. 6:**
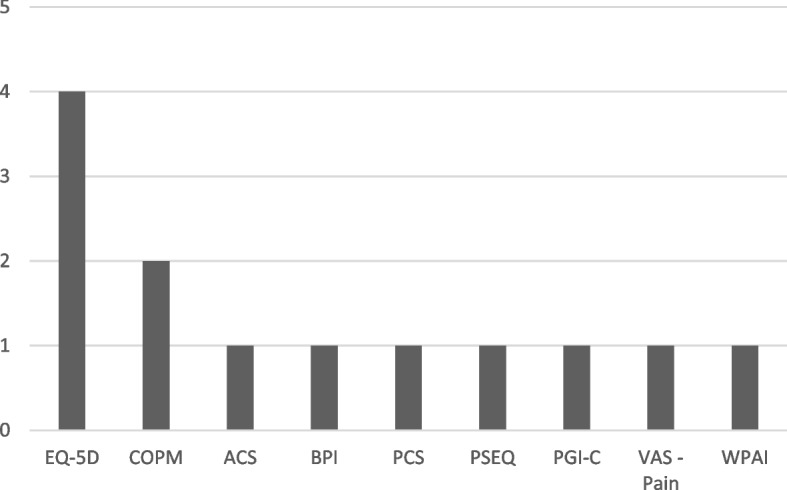
Most frequently used generic Patient Reported Outcome Measures in clinical care. Legend: ACS: Activity Card Sort; BPI: Brief Pain Inventory; COPM: Canadian Occupational Performance Measure; PCS – Pain Catastrophising Scale; PGI-C: Patient Global Impression of Change Scale; PSEQ: Pain Self Efficacy Questionnaire; VAS: Visual Analogue Scale; WPAI: Work Productivity and Activity Impairment Questionnaire

Survey responses were received from all 21 clinical specialties identified. Sixteen (76%) used PROMs for clinical care; all used PROMs to inform patient-provider decisions at the individual patient-level and 5 (31%) to inform care at the service-level. In total, there were 43 PROMs used, 33 (77%) specific and 10 (23%) generic. The median number of PROMs used per clinical specialty was 1 (range: 1 to 4) generic and 3 (range: 1 to 6) specific PROMs. Most clinical specialties measured both body function and activity/participation (81%) and used a specific PROM (75%) with 25% using both a generic and specific PROM (Table 1). The most frequently used generic PROM was the EQ-5D (*n* = 4 clinical specialties, 50% of clinical specialties using a generic PROM), with 3 clinical specialties using the EQ-5D-5L and 1 using the EQ-5D-3L. The Canadian Occupational Performance Measure was the next most commonly used generic PROM (*n* = 2, 25% of clinical specialties using a generic PROM) (Fig. 6). Please refer to Additional files 4 and 5 for a summary of the generic and specific PROMs used for clinical care.

#### Use across clinical specialties

Twenty of 21 (95%) clinical specialties used PROMs for research. The median number of PROMs used per clinical specialty was 4 (range: 1 to 17) generic and 7 (range: 1 to 30) specific PROMs.

Seven of 21 (33%) clinical specialties used PROMs for quality improvement. The median number of PROMs used per clinical specialty was 2 (range: 1 to 4) generic and 2 (range: 1 to 4) specific PROMs.

**Fig. 7:**
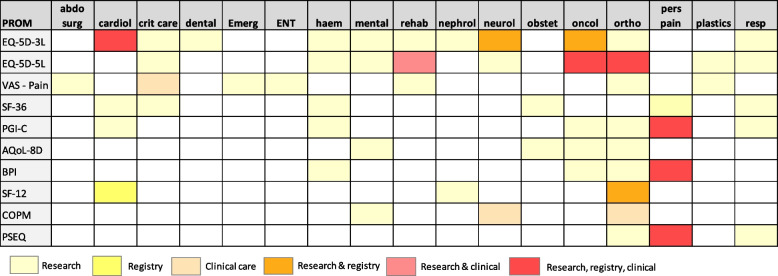
Most frequently used generic PROMs in research projects, quality improvement registries and clinical care across clinical specialties. Legend: abdo surg: abdominal surgery; AQoL-8D: Assessment of Quality of Life-8 Dimensions; BPI: Brief Pain Inventory; COPM: Canadian Occupational Performance Measure; cardiol: cardiology; crit care: critical care; emerg: emergency care; ENT: ear, nose, throat; haem; haematology; PGI-C: Patient Global Impression of Change Scale; PSEQ: Pain Self Efficacy Questionnaire; rehab: rehabilitation; nephrol: nephrology; neurol: neurology; obstet: obstetrics; oncol: oncology; ortho: orthopaedics; pers pain: persistent pain; resp: respiratory; VAS: Visual Analogue Scale; SF-12: 12-Item Short Form Survey; SF-36: 36-Item Short Form Survey

A summary of the most common generic PROMs used across clinical specialties is provided in Fig. 7.

### Practical considerations of PROMs collection

**Fig. 8:**
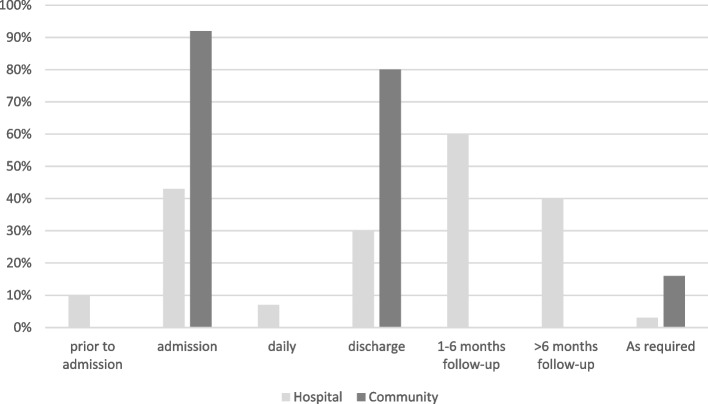
Proportion of Patient Reported Outcome Measures administered during each stage of clinical care in hospital and community settings

Of the 43 PROMs used in clinical care 25 (58%) took ≤ 5 min to administer, 11 (26%) took 6–10 min, 6 (14%) took ≥ 10 min to administer and data were missing for 1 PROM. Data on the mode and timing of collection of PROMs was provided for 55 PROMs, accounting for where an individual PROM was used across multiple clinical services (e.g. inpatients and outpatients). PROMs were mostly exclusively paper-based (*n* = 47, 85%), with very few exclusively electronic (*n* = 2, 4%) or via combination of both modes (*n* = 6, 11%). PROMs were administered face-to-face (*n* = 31, 56%), via a combination of face-to-face and phone (*n* = 15, 27%) or face-to face and email (*n* = 7, 13%). Hospital services typically administered PROMs on admission or preadmission (53%) and at 1–6 months (60%) or greater than 6 months (40%) after discharge from hospital (Fig. 8). Community services typically administered PROMs on admission (92%) and discharge (80%).

An overview of the main findings relating to the purpose, scope and practical considerations of PROM collection is provided in Table 2.

**Table 2:** Summary of main findings relating to the purpose, scope and practical considerations of Patient Reported Outcome Measure (PROM) collection

Recommended step for planning implementation of PROMs	Findings
**Purpose**	• Most PROMs (90%) were used for the purpose of research as an endpoint in a study
	• Most clinical specialties (71%) used PROMs in clinical care to inform patient-provider decisions
**Scope**	• Specific PROMs were more commonly used than generic PROMs in research (83% vs. 65% of projects), quality improvement (69% vs. 54% of registries) and clinical care (75% vs. 50% of clinical specialties)
	• PROMs were mostly used to measure both body function and activity limitation in research (93% of projects), quality improvement (100% of registries), and clinical care (81% of clinical specialties)
	• The EQ-5D was the most commonly used generic PROM across all purposes and clinical specialties
**Practical Considerations**	• PROMs typically took ≤ 5 min to complete (58%)
	• PROMs were mostly paper-based and administered face-to-face (56%) or via a combination of face-to-face and phone (27%)
	• There were differences in timing of PROM administration between hospital and community services; PROMs were typically administered on discharge from community services (80%), while hospital services had longer follow-up, with PROMs administered 1–6 months (60%) or greater than 6 months (40%) following discharge

## Discussion

Our study demonstrates a novel assessment for better understanding the use of PROMs across an entire health organisation. Our results highlight the predominant use of specific PROMs, and that the EQ-5D questionnaires were the most commonly used generic PROMs for all purposes (i.e. research, quality improvement, and clinical care), across a range of clinical specialties. We also identified trends in the method of collection of PROMs in clinical care, revealing key differences between hospital and community services.

Both condition-specific PROMs and generic PROMs have a place in patient reported outcome measurement. Specific PROMs are argued to have greater face validity and responsiveness to change than generic PROMs, and therefore, are better suited to measuring treatment outcomes for specific clinical populations at the individual level [4, 33]. Generic PROMs allow easier comparison across clinical populations and may be better suited to measuring outcomes at an organisational or system level [7]. As such, specific and generic PROMs can be complementary and it is recommended that they are used concurrently to measure outcomes in health systems [33]. Our results indicate that specific and generic PROMs were rarely used together, particularly for quality improvement and clinical care, highlighting a potential gap in the use of PROMs within healthcare organisations.

In addition to measuring outcomes at the organisational level, generic PROMs may also enhance patient-centered care. Health systems are often described as ‘silos of care’ with little collaboration between health providers, both within and between organisations [34, 35]. This environment can impede adoption of patient centered care, which aims to address patient needs and preferences by giving them an active role in decisions regarding their health [36]. This is particularly relevant for patients with multiple chronic health conditions (i.e. multimorbidity) who receive care from multiple providers [34, 37]. Because generic PROMs capture health information that is reflective of patients’ overall health status, and is not specific to the condition or clinical specialty, they have the potential to be shared between healthcare providers. As such, they have the potential to enhance communication of a person’s overall health and reduce unnecessary duplication of assessment between healthcare providers.

The EQ-5D, both three and five-level versions, were identified as generic PROMs with potential for wider implementation across the healthcare organisation. The EQ-5D measures have demonstrated validity and reliability in measuring health status in a broad range of populations and settings, with the EQ-5D-5L demonstrating a lower ceiling effect, higher discriminatory power, and greater validity than the EQ-5D-3L [38–40]. The EQ-5D-5L is therefore the preferred option based on psychometric properties [12, 26, 27]. The acceptability of the EQ-5D, from the perspectives of both clinicians and patients, is less clear. For some clinicians there is some concern that the EQ-5D lacks specificity and therefore, believe that it may not be applicable to their patients or practice [41]. While patients with chronic health conditions believe that it is easy to use and measures domains that are meaningful to them, but may not measure all relevant domains such as fatigue and medication side effects [42, 43]. It may also have limited acceptability in younger patients with chronic health conditions, such as asthma [44]. Further research is required to establish the acceptability of the EQ-5D in the full spectrum of healthcare professions and patient populations.

In addition to the psychometric properties, content and acceptability, it is important to consider the practicalities of administering the PROM to minimise responder and administrator burden and ensure adequate data is captured (i.e. high completion rate) [27]. Our results showed that over 80% of PROMs administered at the health service took 10 min or less to complete. This may provide an indication of the current acceptable length of PROM completion within the health organisation, but would need to be confirmed through engagement with patients and clinicians. Very few PROMs were administered electronically, which indicates a possible lack of information technology structures or siloed approaches established by clinicians without organisational direction. This is a significant barrier to wider implementation of PROMs across the organisation and would need to be addressed to streamline data collection and reduce the cost and burden of collection [12, 26, 27]. We also found that hospital and community-based services had distinct patterns in timing of administration; hospital services administered PROMs on admission/pre-admission and 1–6 months post discharge from hospital, while community services administered PROMs on admission to and discharge from the service. These timings of PROM administration have the potential to coincide for patients who are attending both hospital outpatient and community-based clinics (i.e. patients referred to community rehabilitation following surgery). Therefore, if PROMs were to be implemented across an organisation the timing of administration will need to balance the needs of individual services and the need to reduce responder burden/duplication.

The findings from this study have important implications for our future program of work. First, our findings from this study (i.e. study 1) indicate that the EQ-5D may be an acceptable generic PROM and already has buy-in from the clinical specialties for the purpose of research, quality improvement and clinical care. Study 2 and 3 will explore clinicians’ and patients’ acceptability of using the EQ-5D in routine care, and whether the EQ-5D captures aspects of health that are relevant to their care. Our findings also provide useful insights on how PROMs are currently administered to patients. In study 4 we will engage with patients to codesign a PROMs collection system, and establish whether the current processes for PROMs collection meet their preferences. An important component of this will be establishing how best we can integrate and support electronic collection systems. More generally, our finding that a high proportion of clinical specialties are using mostly specific PROMs to inform their clinical care may act as a barrier to implementation of a generic PROM across the organisation. Clear messaging on the intention for a generic PROM to complement, and not replace, the use of a specific PROM will be required, as will training on how to use generic and specific PROMs in a complementary manner.

Our study demonstrates a comprehensive method, that could be adopted by other healthcare organisations, for assessing the use of PROMs across a healthcare organisation to understand the pre-implementation context for routine PROMs collection. A strength of our study is the use of established research methods, including systematic literature search, audit, and surveys. We also used pre-determined definitions and processes for establishing the scope of PROMs [5–8, 27, 29]. A limitation is that our results may not specifically reflect the use of PROMs more broadly in other healthcare organisations. While we used multiple methods to establish the use of PROMs for research and quality improvement, we were limited to use of self-report surveys to assess the use of PROMs in clinical care and may have failed to identify some PROMs used in clinical care. However, we had significant engagement with program managers and directors who assisted in the process of identifying clinical leaders, increasing the likelihood that we captured the majority of PROMs used in clinical practice.

## Conclusions

We have presented a process for developing a comprehensive understanding of the current use of PROMs across an entire healthcare organisation. Using this approach, we have elicited critical preliminary information on the purpose, scope and practical considerations of PROMs collection; information that is important for guiding organisational wide implementation of PROMs collection. Within the context of our healthcare organisation, PROMs were mostly collected for the purpose of research, however, the majority of clinical specialties also used PROMs to inform their clinical care; while specific PROMs were more commonly used than generic PROMs, the EQ-5D was the most commonly used PROM across the organisation; and PROMs were typically paper-based and administered face-to-face. Future work will determine clinician and patient acceptability of the EQ-5D to determine its potential for implementation across the organisation, and co-design a system for the collection and use of PROMs across the healthcare organisation.

## Supplementary Information

**Additional file 1. **Literature search strategy 

**Additional file 2. **Survey questions 

**Additional file 3.** Literature search results

**Additional file 4. **Summary of generic patient reported outcome measures 

**Additional file 5. **Summary of specific patient reported outcome measures

## Data Availability

All data generated or analysed during this study are included in this published article [and its supplementary information files].

## Abbreviations

PROM: patient reported outcome measure
