# Supplementary material for: A landscape assessment of the use of patient reported outcome measures in research, quality improvement and clinical care across a healthcare organisation
Source: BMC Health Serv Res. 2023 Jan 27;23:94. doi: 10.1186/s12913-023-09050-1 (PMC9883937; doi:10.1186/s12913-023-09050-1)
Supplement: Supplementary file 1 — Additional file 1. Literature search strategy [file 12913_2023_9050_MOESM1_ESM.docx]

**Additional File 1**. Literature search strategy

| **Search strategy** |
| --- |
| (Peninsula Health OR Frankston Hospital OR Rosebud Hospital OR Golf Links Road Rehabilitation Centre OR The Mornington Centre OR Frankston Community Health OR Hastings Community Health OR Mornington Community Health OR Rosebud Community Health OR Bayview House OR Peninsula Mental Health Service) |
| AND |
| Australia |

**ADDITIONAL FILE DETAILS**

File name: Additional file 1

File format: .docx

Title of data: Literature Search Strategy

Description of data: An outline of the literature search strategy used to identify research projects that had used patient reported outcome measures.
