# Supplementary material for: A landscape assessment of the use of patient reported outcome measures in research, quality improvement and clinical care across a healthcare organisation
Source: BMC Health Serv Res. 2023 Jan 27;23:94. doi: 10.1186/s12913-023-09050-1 (PMC9883937; doi:10.1186/s12913-023-09050-1)
Supplement: Supplementary file 2 — Additional file 2. Survey questions [file 12913_2023_9050_MOESM2_ESM.docx]

**Additional File 2**. Survey questions

| **Question** | **Response** |
| --- | --- |
| What clinical program do you work in? | Free text |
| What clinical specialty do you work in? | Free text |
| Does your clinical program use PROMs? | Yes/No |
| What is the name of PROM(s) | Free text |
| What purpose is PROM(s) used for?* | Clinical care / Contribute to registry (i.e. quality improvement)/ Research/Other  (Free text response available) |
| When is PROM(s) administered to patients?* | Admission/Discharge/Follow-up/Other (Free text response available) |
| How is PROM administered to patients?* | Email/Web-based/Paper-based/Face-to-Face/Phone/Mail/Tablet/Phone/Other  (Free text response available) |
| How long does it take to administer PROM(s)? | Free text response |

PROM – Patient Reported Outcome Measure; *can select more than one response.

**ADDITIONAL FILE DETAILS**

File name: Additional file 2

File format: .docx

Title of data: Survey Questions

Description of data: The survey that was administered to health professionals to identify patient reported outcome measures used in research projects, data registries and clinical care.
