# Supplementary material for: A landscape assessment of the use of patient reported outcome measures in research, quality improvement and clinical care across a healthcare organisation
Source: BMC Health Serv Res. 2023 Jan 27;23:94. doi: 10.1186/s12913-023-09050-1 (PMC9883937; doi:10.1186/s12913-023-09050-1)
Supplement: Supplementary file 3 — Additional file 3. Literature search results [file 12913_2023_9050_MOESM3_ESM.docx]

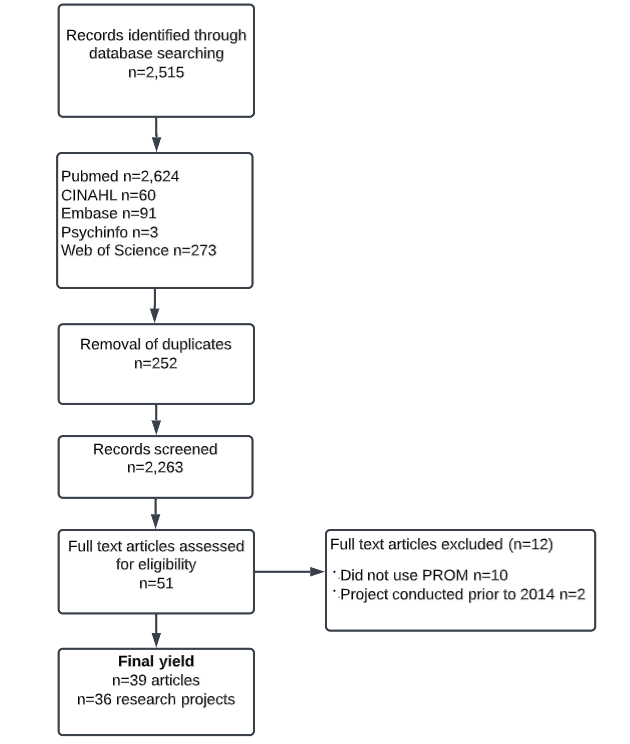


**Additional File 3**. Literature search results

**ADDITIONAL FILE DETAILS**

File name: Additional file 3

File format: .docx

Title of data: Literature search results

Description of data: A flow chart displaying the results from the literature search, including the number of records obtained from each database and the final yield of articles.
