# Supplementary material for: A landscape assessment of the use of patient reported outcome measures in research, quality improvement and clinical care across a healthcare organisation
Source: BMC Health Serv Res. 2023 Jan 27;23:94. doi: 10.1186/s12913-023-09050-1 (PMC9883937; doi:10.1186/s12913-023-09050-1)
Supplement: Supplementary file 4 — Additional file 4. Summary of generic patient reported outcome measures [file 12913_2023_9050_MOESM4_ESM.docx]

**Additional File 4**. Summary of generic patient reported outcome measures

| **Patient reported outcome measure** | **Research** | | **Quality**  **improvement** | | **Clinical Care** | |
| --- | --- | --- | --- | --- | --- | --- |
|  | Projects  n | List of clinical specialties | Registries  n | List of clinical specialties | Clinical  specialties  n | List of clinical specialties |
| 12-Item Short Form Survey (SF-12) | 2 | Nephrology  Orthopaedics | 2 | Cardiology  Orthopaedics | 0 | - |
| 36-Item Short Form Survey (SF-36) | 9 | Cardiology  Critical care  Haematology  Obstetrics  Persistent pain  Respiratory | 0 | - | 0 | - |
| Activity Card Sort (ACS) | 0 | - | 0 | - | 1 | Neurology |
| Arizona Sexual Experiences Scale (ASEX) | 1 | Mental health | 0 | - | 0 | - |
| Assessment of Quality of Life-4 Dimensions (AQoL-4D) | 2 | Orthopaedics | 0 | - | 0 | - |
| Assessment of Quality of Life-8 Dimensions  (AQoL-8D) | 5 | Mental health  Obstetrics  Oncology  Orthopaedics | 0 | - | 0 | - |
| Beck's Hopelessness Scale (BHS) | 1 | Mental health | 0 | - | 0 | - |
| Brief Fatigue Inventory (BFI) | 1 | Oncology | 0 | - | 0 | - |
| Brief Pain Inventory (BPI) | 8 | Haematology  Oncology  Persistent pain | 1 | Persistent pain | 1 | Persistent pain |
| **Patient reported outcome measure** | **Research** | | **Quality**  **improvement** | | **Clinical Care** | |
|  | Projects  n | List of clinical specialties | Registries  n | List of clinical specialties | Clinical  specialties  n | List of clinical specialties |
| Brief Resilient Coping Scale (BRCS) | 1 | Mental health | 0 | - | 0 | - |
| Brief Symptom Inventory (BSI) | 1 | Oncology | 0 | - | 0 | - |
| Canadian Occupational Performance Measure (COPM) | 1 | Mental health | 0 | - | 2 | Neurology  Orthopaedics |
| Center for Epidemiologic Studies Depression Scale (CES-D) | 1 | Mental health | 0 | - | 0 | - |
| Consensus Sleep Diary (CSD) | 1 | Mental health | 0 | - | 0 | - |
| Death Anxiety Questionnaire (DAQ) | 1 | Geriatrics | 0 | - | 0 | - |
| Distress Tolerance Scale (DTS) | 1 | Mental health | 0 | - | 0 | - |
| Epworth Sleepiness Scale (ESS) | 1 | Geriatrics | 0 | - | 0 | - |
| EQ-5D-5L | 31 | Critical care  Haematology  Mental health  Neurology  Oncology  Orthopaedics  Plastics  Rehabilitation  Respiratory | 2 | Oncology  Orthopaedics | 3 | Oncology  Orthopaedics  Rehabilitation |
| EQ-5D-3L | 25 | Cardiology  Critical care  Dental | 3 | Cardiology  Neurology  Orthopaedics | 1 | Cardiology |
| **Patient reported outcome measure** | **Research** | | **Quality**  **improvement** | | **Clinical Care** | |
|  | Projects  n | List of clinical specialties | Registries  n | List of clinical specialties | Clinical  specialties  n | List of clinical specialties |
| EQ-5D-3L (cont.) |  | Haematology  Mental health  Nephrology  Neurology  Oncology  Orthopaedics  Rehabilitation  Respiratory |  |  |  |  |
| Frenchay Activities Index (FAI) | 2 | Geriatrics  Orthopaedics | 0 | - | 0 | - |
| Generalised Self-Efficacy Scale (GSES) | 1 | Mental health | 0 | - | 0 | - |
| Intrinsic Spirituality Scale (ISS) | 1 | Geriatrics | 0 | - | 0 | - |
| Nottingham Extended Activities of Daily Living Scale (NEADL) | 1 | Neurology | 0 | - | 0 | - |
| Numerical Pain Rating Scale (NPRS) | 3 | ENT  Orthopaedics | 1 | Orthopaedics | 0 | - |
| Pain Catastrophising Scale (PCS) | 2 | Rehabilitation  Persistent pain | 1 | Persistent pain | 1 | Persistent pain |
| Pain Self Efficacy Questionnaire (PSEQ) | 2 | Persistent pain  Respiratory | 1 | Persistent pain | 1 | Persistent pain |
| Patient Global Impression of Change Scale  (PGI-C) | 12 | Cardiology  Haematology  Oncology  Orthopaedics  Persistent pain  Respiratory | 1 | Persistent pain | 1 | Persistent pain |
| **Patient reported outcome measure** | **Research** | | **Quality**  **improvement** | | **Clinical Care** | |
|  | Projects  n | List of clinical specialties | Registries  n | List of clinical specialties | Clinical  specialties  n | List of clinical specialties |
| Patient Global Impression of Severity Scale  (PGI-S) | 4 | Cardiology  Respiratory | 0 | - | 0 | - |
| Personal Well-Being Index-Adult (PWI-A) | 2 | Neurology  Obstetrics | 0 | - | 0 | - |
| Pittsburgh Sleep Quality Index (PSQI) | 1 | Geriatrics | 0 | - | 0 | - |
| Proactive Personality Scale (PPS) | 1 | Mental health | 0 | - | 0 | - |
| Quality of Life Enjoyment and Satisfaction Questionnaire - Short Form (Q-LES-Q-SF) | 1 | Mental health | 0 | - | 0 | - |
| Sheehan Disability Scale (SDS) | 2 | Mental health | 0 | - | 0 | - |
| Sleep-50 Questionnaire | 1 | Mental health | 0 | - | 0 | - |
| Ten-Item Personality Inventory  (TIPI) | 1 | Geriatrics | 0 | - | 0 | - |
| The Friendship Scale (FS) | 2 | Geriatrics | 0 | - | 0 | - |
| University of California, Los Angeles (UCLA) Loneliness Scale | 1 | Mental health | 0 | - | 0 | - |
| Visual Analogue Scale (VAS) – Fatigue | 1 | Haematology | 0 | - | 0 | - |
| Visual Analogue Scale (VAS) – Pain | 8 | Abdominal surgery  Emergency  ENT  Rehabilitation  Orthopaedics  Persistent pain | 0 | - | 1 | Critical care |
| **Patient reported outcome measure** | **Research** | | **Quality**  **improvement** | | **Clinical Care** | |
|  | Projects  n | List of clinical specialties | Registries  n | List of clinical specialties | Clinical  specialties  n | List of clinical specialties |
| Visual Analogue Scale (VAS) - Quality of Sleep | 1 | Critical care | 0 | - | 0 | - |
| Work Productivity and Activity Impairment Questionnaire (WPAI) | 5 | Orthopaedics  Respiratory | 1 | Respiratory | 1 | Respiratory |
| World Health Organisation Quality of Life - BREF (WHOQOL-BREF) | 2 | ENT  Mental health | 0 | - | 0 | - |
| World Health Organization Disability Assessment Schedule 2.0 (WHODAS 2.0) | 0 | - | 1 | Orthopaedics | 0 | - |

**ADDITIONAL FILE DETAILS**

File name: Additional file 4

File format: .docx

Title of data: Summary of generic patient reported outcome measures

Description of data: A table that summarises all the generic patient reported outcome measures that were identified during the mapping process, why they were administered (i.e., research, quality improvement, clinical care), and the clinical specialties that used each generic patient reported outcome.
