# Supplementary material for: A landscape assessment of the use of patient reported outcome measures in research, quality improvement and clinical care across a healthcare organisation
Source: BMC Health Serv Res. 2023 Jan 27;23:94. doi: 10.1186/s12913-023-09050-1 (PMC9883937; doi:10.1186/s12913-023-09050-1)
Supplement: Supplementary file 5 — Additional file 5. Summary of specific patient reported outcome measures [file 12913_2023_9050_MOESM5_ESM.docx]

**Additional File 5**. Summary of specific patient reported outcome measures

| **PROM** | **Research** | **Quality**  **improvement** | **Clinical Care** |
| --- | --- | --- | --- |
|  | Projects  n | Registries  n | Clinical  specialties  n |
| *Alcohol and drug* | | | |
| Alcohol Use Disorders Identification Test (AUDIT) | 2 | 0 | 1 |
| Alcohol-related problems survey (A-ARPS) | 3 | 0 | 1 |
| Drug Use Disorders Identification Test (DUDIT) | 1 | 0 | 1 |
| Kessler Psychological Distress Scale (K10) | 1 | 0 | 0 |
| Modified Mini Screen (MMS) | 1 | 0 | 0 |
| *Cardiology* | | | |
| Chronic Heart Failure Questionnaire Self- Administered Standardized Format  (CHQ-SAS) Dyspnoea score | 1 | 0 | 0 |
| Kansas City Cardiomyopathy Questionnaire (KCCQ-12) | 1 | 0 | 0 |
| *Geriatrics* | | | |
| Ambulatory Self-Confidence Questionnaire (ASCQ) | 2 | 0 | 0 |
| Dizziness Handicap Inventory (DHI) | 0 | 0 | 1 |
| Lubben Social Network Scale Abbreviated (LSNS-6) | 2 | 0 | 0 |
| Modified Falls Efficacy Scale (MFES) | 0 | 0 | 1 |
| Mood and Physical Symptoms Scale (MPSS) | 1 | 0 | 0 |
| Patient Health Questionnaire (PHQ-9) | 1 | 0 | 0 |
| Phone-FITT | 3 | 0 | 0 |
| Post Discharge Coping Difficulty Scale (PDCDS) | 1 | 0 | 0 |
| Readiness for Hospital Discharge (RHDS) | 1 | 0 | 0 |
| Urinary Distress Inventory (UDI-6) | 1 | 0 | 0 |
| *Haematology* | | | |
| Depression, Anxiety and Stress Scales (DASS-21) | 1 | 0 | 0 |
| **PROM** | **Research** | **Quality**  **improvement** | **Clinical Care** |
|  | Projects  n | Registries  n | Yes/no |
| *Haematology (cont.)* | | | |
| European Organisation for Research and Treatment of Cancer Quality of Life Questionnaire (EORTC QLQ C30) | 8 | 0 | No |
| European Organisation for Research and Treatment of Cancer Quality of Life Questionnaire Chronic Lymphocytic Leukaemia Module (EORTC QLQ CLL17) | 1 | 0 | No |
| European Organisation for Research and Treatment of Cancer Quality of Life Questionnaire Myeloma Module (EORTC QLQ MY20) | 1 | 0 | No |
| Functional Assessment of Cancer Therapy - General (FACT-G) | 2 | 0 | No |
| Functional Assessment of Cancer Therapy - Leukemia (FACT-leu) | 3 | 0 | No |
| Functional Assessment of Chronic Illness Treatment - Fatigue (FACIT-F) | 3 | 0 | No |
| Godin-Shephard Leisure-Time Physical Activity Questionnaire (GSLTPAQ) | 1 | 0 | No |
| The Modified Myelofibrosis Symptom Assessment Form (MFSAF) | 2 | 0 | No |
| *Mental health* | | | |
| Altman Self Rating Mania (ASRM) | 1 | 0 | No |
| Bayer Activities of Daily Living (B-ADL) | 1 | 0 | No |
| Behaviour and Symptom Identification Scale (BASIS-32) | 1 | 0 | Yes |
| Dementia Quality of Life Instrument (DEM-QOL) | 1 | 0 | No |
| Depression, Anxiety and Stress Scales (DASS-21) | 1 | 0 | No |
| Dysfunctional Beliefs and Attitudes about Sleep (DBAS-10) | 1 | 0 | No |
| Everyday Cognition (Ecog) Scale | 1 | 0 | No |
| Generalised Anxiety Disorder-7 (GAD-7) | 1 | 0 | No |
| Geriatric Anxiety Inventory SF (GAI-SF) | 1 | 0 | No |
| Geriatric Depression Scale (GDS) | 1 | 0 | No |
| Hospital Anxiety and Depression Scale (HADS) | 2 | 0 | No |
| Illness Cognition Questionnaire (ICQ) | 1 | 0 | No |
| Insomnia Severity Index (ISI) | 1 | 0 | No |
| Internalised Stigma of Mental Illness Scale (ISMI) | 1 | 0 | No |
| **PROM** | **Research** | **Quality**  **improvement** | **Clinical Care** |
|  | Projects  n | Registries  n | Yes/no |
| *Mental health (cont.)* | | | |
| Kessler Psychological Distress Scale (K10) | 1 | 0 | No |
| Massachusetts General Hospital Cognitive and Physical Functioning Questionnaire (CPFQ) | 1 | 0 | No |
| Mood Disorder Questionnaire (MDQ) | 1 | 0 | No |
| Multifactorial Meta Cognition Questionnaire (MMQ) - Ability | 1 | 0 | No |
| Patient Health Questionnaire (PHQ-9) | 2 | 0 | No |
| Process of Recovery Questionnaire (QPR) | 1 | 0 | No |
| Psychosis Attachment Measure (PAM) | 1 | 0 | No |
| Quality of Life in Alzheimer's Disease (QOL-AD) | 1 | 0 | No |
| Recovery Style Questionnaire (RSQ) | 1 | 0 | No |
| Revised Memory and Behaviour Checklist (RMBC) | 1 | 0 | No |
| Schizophrenia Hope Scale-9 (SHS-9) | 1 | 0 | No |
| Self-report Strengths and Difficulties Questionnaire (SDQ) | 0 | 0 | Yes |
| Social Functioning Scale (SFS) | 1 | 0 | No |
| Subjective Experience of Psychosis Scale (SEPS) | 1 | 0 | No |
| Zarit Burden Interview (ZBI) | 1 | 0 | No |
| Zarit Burden Interview Short Version (ZBI-12) | 1 | 0 | No |
| *Nephrology* |  |  |  |
| Integrative Palliative Care Outcome Scale - Renal (iPOS renal) | 1 | 0 | Yes |
| *Neurology* |  |  |  |
| Fatigue Assessment Scale (FAS) | 0 | 0 | Yes |
| Health Education Impact Questionnaire (HEiQ) | 1 | 0 | No |
| Hospital Anxiety and Depression Scale (HADS) | 1 | 0 | Yes |
| Longer Unmet Needs After Stroke (LUNS) Questionnaire | 1 | 0 | No |
| Modified Falls Efficacy Scale (MFES) | 1 | 0 | No |
| Multiple Sclerosis Quality of Life Questionnaire (MSQOL-54) | 1 | 0 | No |
| **PROM** | **Research** | **Quality**  **improvement** | **Clinical Care** |
|  | Projects  n | Registries  n | yes/no |
| *Neurology (cont.)* | | | |
| Non-Motor Symptom Scale (NMSS) | 0 | 0 | Yes |
| Parkinson's Disease Questionnaire-39 (PDQ-39) | 1 | 0 | Yes |
| Stroke Self Efficacy Questionnaire (SSEQ) | 1 | 0 | No |
| Stroke Aphasic Depression Questionnaire (SADQ) | 0 | 0 | Yes |
| *Obstetrics* | | | |
| Antenatal Risk Questionnaire (ANRQ) | 0 | 0 | Yes |
| Depression, Anxiety and Stress Scales (DASS-21) | 2 | 0 | No |
| Edinburgh Postnatal Depression Scale (EPDS) | 1 | 0 | Yes |
| State Trait Anxiety Inventory (STAI) | 1 | 0 | No |
| *Oncology* | | | |
| Cancer Survivors Unmet Needs Measure (CaSUN) | 1 | 0 | No |
| Distress Thermometer (DT) | 1 | 0 | No |
| Duke Activity Status Index (DASI) | 1 | 0 | No |
| European Organisation for Research and Treatment of Cancer Quality of Life Questionnaire Breast Cancer Module (EORTC QLQ BR23) | 3 | 0 | No |
| European Organisation for Research and Treatment of Cancer Quality of Life Questionnaire (EORTC QLQ C30) | 22 | 1 | No |
| European Organisation for Research and Treatment of Cancer Quality of Life Questionnaire Colorectal Cancer Module (EORTC QLQ CR38) | 1 | 0 | No |
| European Organisation for Research and Treatment of Cancer Quality of Life Questionnaire Endometrial Cancer Module (EORTC QLQ EN24) | 1 | 0 | No |
| European Organisation for Research and Treatment of Cancer Quality of Life Questionnaire Gastric Cancer Module (EORTC STO 22) | 2 | 0 | No |
| European Organisation for Research and Treatment of Cancer Quality of Life Questionnaire Oesophageal Cancer Module (EORTC OES 18) | 1 | 0 | No |
| **PROM** | **Research** | **Quality**  **improvement** | **Clinical Care** |
|  | Projects  n | Registries  n | yes/no |
| *Oncology (cont.)* | | | |
| European Organisation for Research and Treatment of Cancer Quality of Life Questionnaire Ovarian Cancer Module (EORTC QLQ OV28) | 2 | 0 | No |
| European Organisation for Research and Treatment of Cancer Quality of Life Questionnaire Palliative Care Module (EORTC QLQ C15 PAL) | 1 | 0 | No |
| European Organisation for Research and Treatment of Cancer Quality of Life Questionnaire Updated Breast Cancer Module (EORTC QLQ BR45) | 1 | 0 | No |
| Expanded Prostate Cancer Index Composite-26 (EPIC-26) | 0 | 1 | No |
| Functional Assessment of Cancer Therapy - Bladder - Cystectomy (FACT-BI-CYS) | 1 | 0 | No |
| Functional Assessment of Cancer Therapy - Bladder (FACT-BI) | 2 | 0 | No |
| Functional Assessment of Cancer Therapy - Breast (FACT-B) | 1 | 0 | No |
| Functional Assessment of Cancer Therapy - General (FACT-G) | 2 | 0 | No |
| Functional Assessment of Cancer Therapy - Lymphoma (FACT-Lym) | 4 | 0 | No |
| Functional Assessment of Cancer Therapy - Taxane (FACT-Taxane) | 1 | 0 | No |
| Functional Assessment of Cancer Therapy/Gynaecologic Oncology Group - Neurotoxicity (FACT/GOG-NTX) | 1 | 0 | No |
| Hospital Anxiety and Depression Scale (HADS) | 1 | 0 | No |
| International Prostate Symptom Score (IPSS) | 1 | 0 | No |
| Patient Generated Subjective Global Assessment (PG-SGA) | 1 | 0 | No |
| Patient Health Questionnaire (PHQ-9) | 1 | 0 | No |
| Patient-Reported Outcomes version of the Common Terminology Criteria for Adverse Events (PRO-CTCAE™) | 1 | 0 | No |
| Supportive Care Needs Scale (SCNS) | 1 | 0 | No |
| *Orthopaedics* |  |  |  |
| Ambulatory Self-Confidence Questionnaire (ASCQ) | 1 | 0 | No |
| **PROM** | **Research** | **Quality**  **improvement** | **Clinical Care** |
|  | Projects  n | Registries  n | Yes/no |
| *Orthopaedics (cont.)* | | | |
| American Academy of Orthopaedic Surgeons (AAOS) Foot and Ankle Questionnaire | 1 | 0 | No |
| American Shoulder and Elbow Surgeons Standardized Shoulder Assessment Form (ASES) | 1 | 0 | No |
| Depression, Anxiety and Stress Scales (DASS-21) | 1 | 0 | No |
| Disabilities of the arm, shoulder, hand (DASH) | 1 | 0 | Yes |
| Faces Pain Scale (FPS) | 1 | 0 | No |
| Hip Dysfunction and Osteoarthritis Outcome Score (HOOS) | 2 | 0 | No |
| ICEpop Capability Measure for Older People (ICECAP-O) | 1 | 0 | No |
| Kessler Psychological Distress Scale (K10) | 3 | 0 | No |
| Knee Injury and Osteoarthritis Outcome Score (KOOS) | 1 | 0 | No |
| Lower Extremity Functional Scale (LEFS) | 0 | 0 | Yes |
| Manchester-Oxford Foot Questionnaire (MOXFQ) | 1 | 0 | No |
| Modified Falls Efficacy Scale (MFES) | 1 | 0 | No |
| Oxford Hip Score (OHS) | 3 | 0 | No |
| Oxford Knee Score (OKS) | 2 | 0 | No |
| Oxford Shoulder Score (OSS) | 2 | 0 | Yes |
| Parenting Disability Index (PDI) | 1 | 0 | No |
| Patient-Rated Wrist Evaluation (PRWE) | 0 | 0 | Yes |
| Pediatric Quality of Life Inventory (PedsQL) | 0 | 1 | No |
| Quick Disabilities of Arm, Shoulder and Hand (QuickDASH) | 1 | 0 | No |
| The Multi-attribute Arthritis Prioritisation Tool (MAPT) | 0 | 0 | Yes |
| The Oxford Foot Questionnaire for Children (OxAFQ-C) | 2 | 0 | No |
| Upper Extremity Function Index (UEFI) | 0 | 0 | Yes |
| Victorian Institute of Sport Assessment - Gluteal Questionnaire (VISA-G) | 1 | 0 | No |
| Visual Analogue Scale (VAS) - Limp | 1 | 0 | No |
| **PROM** | **Research** | **Quality**  **improvement** | **Clinical Care** |
|  | Projects  n | Registries  n | Yes/no |
| *Orthopaedics (cont.)* | | | |
| Visual Analogue Scale (VAS) - Normality of the joint | 1 | 0 | No |
| Western Ontario and McMaster Universities Osteoarthritis Index (WOMAC) | 1 | 0 | No |
| Workplace Activity and Limitation Scale (WALS) | 1 | 0 | No |
| Children's Leisure Activities Study Survey (CLASS) | 1 | 0 | No |
| *Palliative care* | | | |
| Palliative Care Outcomes Collaboration Symptom Assessment Scale (PCOC-SAS) | 0 | 0 | Yes |
| *Persistent pain* | | | |
| Depression, Anxiety and Stress Scales (DASS-21) | 1 | 1 | Yes |
| *Pelvic health* | | | |
| Australian Pelvic Floor Questionnaire (APFQ) | 0 | 0 | Yes |
| Pelvic Floor Distress Inventory (PFDI) | 0 | 0 | Yes |
| Pelvic Floor Impact Questionnaire (PFIQ) | 0 | 0 | Yes |
| *Plastics* | | | |
| Boston Carpel Tunnel Questionnaire (BCTQ) | 1 | 0 | No |
| Breast Q (mastectomy) | 1 | 1 | Yes |
| British Society for Surgery of the Hand survey | 1 | 0 | No |
| Patient Evaluation Measure (PEM) | 1 | 0 | No |
| The Southampton Dupuytren's Scoring Scheme (SDSS) | 2 | 1 | Yes |
| Unité Rhumatologique des Affections de la Main (URAM) | 2 | 1 | Yes |
| Wound-QoL | 1 | 0 | No |
| *Respiratory* | | | |
| Asthma Control Questionnaire (ACQ) | 12 | 2 | Yes |
| Asthma Control Test (ACT) | 2 | 0 | No |
| Asthma Quality of Life Questionnaire (AQLQ) | 10 | 2 | Yes |
| Asthma Quality of Life Questionnaire-Standardised (AQLQ) | 0 | 0 | Yes |
| **PROM** | **Research** | **Quality**  **improvement** | **Clinical Care** |
|  | Projects  n | Registries  n | Yes/no |
| *Respiratory (cont.)* | | | |
| Asthma Symptom Utility Index (ASUI) | 1 | 0 | No |
| Baseline/Transitional Dyspnoea Index (BDI/TDI) | 1 | 0 | No |
| Chronic Airways Assessment Test (CAAT) | 1 | 0 | No |
| Chronic Respiratory Questionnaire (CRQ) | 1 | 0 | No |
| COPD Assessment in Primary Care to Identify Undiagnosed Respiratory Disease & Exacerbation Risk (CAPTURE) | 1 | 0 | No |
| COPD Assessment Test (CAT) | 8 | 0 | No |
| Dysponea-12 Questionnaire (D-12) | 1 | 0 | No |
| Global Initiative for Asthma (GINA) assessment of asthma control | 1 | 1 | Yes |
| Hospital Anxiety and Depression Scale (HADS) | 3 | 1 | Yes |
| Kings Brief Interstitial Lung Disease (KBILD) Questionnaire | 1 | 0 | No |
| Leicester Cough Questionnaire (LCQ) | 1 | 0 | No |
| Modified Borg Dyspnoea Scale | 1 | 0 | No |
| Modified Medical Research Council (mMRC) Dyspnoea Scale | 2 | 0 | No |
| MOS Sleep Scale | 1 | 0 | No |
| Quality of Life for Bronchiectasis (QOL-B) | 2 | 0 | No |
| Sino-Nasal Outcome Test (SNOT-22) | 2 | 0 | No |
| St George Respiratory Questionnaire (SGRQ) | 14 | 0 | No |
| Standardised Rhinoconjunctivitis Quality of Life Questionnaire (RQLQ) | 1 | 0 | No |
| The Exacerbations of Chronic Pulmonary Disease Tool (EXACT) | 1 | 0 | No |
| University of California San Diego (UCSD) Shortness of Breath Questionnaire | 3 | 0 | No |
| Visual Analogue Scale (VAS) - Cough Severity | 1 | 0 | No |

PROM – Patient Reported Outcome Measure.

**ADDITIONAL FILE DETAILS**

File name: Additional file 5

File format: .docx

Title of data: Summary of specific patient reported outcome measures

Description of data: A table that summarises all the specific patient reported outcome measures that were identified during the mapping process, why they were administered (i.e., research, quality improvement, clinical care), and the clinical specialties that used each specific patient reported outcome.
